# Electrochemotherapy Using Doxorubicin and Nanosecond Electric Field Pulses: A Pilot in Vivo Study

**DOI:** 10.3390/molecules25204601

**Published:** 2020-10-09

**Authors:** Vitalij Novickij, Veronika Malyško, Augustinas Želvys, Austėja Balevičiūtė, Auksė Zinkevičienė, Jurij Novickij, Irutė Girkontaitė

**Affiliations:** 1 Faculty of Electronics, Vilnius Gediminas Technical University, 03227 Vilnius, Lithuania; veronika.malysko@vgtu.lt (V.M.); jurij.novickij@vgtu.lt (J.N.); 2 Department of Immunology, State Research Institute Centre for Innovative Medicine, 08406 Vilnius, Lithuania; augustinas.zelvys@gmail.com (A.Ž.); baleviciuteausteja@gmail.com (A.B.); aukse.zinkeviciene@imcentras.lt (A.Z.); irute.girkontaite@imcentras.lt (I.G.)

**Keywords:** electrochemotherapy, electroporation, drugs, tumors

## Abstract

Pulsed electric field (PEF) is frequently used for intertumoral drug delivery resulting in a well-known anticancer treatment—electrochemotherapy. However, electrochemotherapy is associated with microsecond range of electrical pulses, while nanosecond range electrochemotherapy is almost non-existent. In this work, we analyzed the feasibility of nanosecond range pulse bursts for successful doxorubicin-based electrochemotherapy in vivo. The conventional microsecond (1.4 kV/cm × 100 µs × 8) procedure was compared to the nanosecond (3.5 kV/cm × 800 ns × 250) non-thermal PEF-based treatment. As a model, Sp2/0 tumors were developed. Additionally, basic current and voltage measurements were performed to detect the characteristic conductivity-dependent patterns and to serve as an indicator of successful tumor permeabilization both in the nano and microsecond pulse range. It was shown that nano-electrochemotherapy can be the logical evolution of the currently established European Standard Operating Procedures for Electrochemotherapy (ESOPE) protocols, offering better energy control and equivalent treatment efficacy.

## 1. Introduction

Electroporation is a phenomenon of reversible [1,2] or irreversible [3] permeabilization of biological cells, which is triggered by cell polarization in pulsed electric fields (PEF). Among the most established applications of electroporation is the treatment of cancer [4]. This allows the minimization of the dosage of chemotherapeutic drugs [5,6] and/or adverse effects during the ablation of the tumor [7]. Consequently, the combination of chemotherapeutic drugs and electroporation was coined electrochemotherapy and is widely used in clinics [8]. Nevertheless, electrochemotherapy is associated with microsecond range of electrical pulses [9,10,11,12,13], while nanosecond range electrochemotherapy is almost non-existent [14,15,16]. At the current state, nanosecond pulses are typically used for tissue ablation, or in other words, drug-free anticancer therapy [17,18].

However, in recent years, the interest in shorter pulse electroporation has increased dramatically. It was determined that shorter pulses potentially enable a more uniform electric field distribution in non-homogeneous tissue [19], better energy control due to shorter pulses and non-thermal treatment [20], less muscle contractions [21] and also trigger a variety of bioelectric phenomena including the generation of reactive oxygen species [22,23,24] or even the manipulation of cell death type [25]. Lastly, less electrochemical reactions are induced at the electrode interface, which may have less adverse effects on drug pharmokinetics [26]. Almost all of these improvements are desired in the field of electrochemotherapy as well, which is currently dominated by European Standard Operating Procedures for Electrochemotherapy (ESOPE) microsecond range protocols. At the same time, the pulse parameters represent only half of the electrochemotherapy treatment components, while the other half depends on the chemotherapeutic drug. Currently, bleomycin and cisplatin are dominating the field [9,27,28,29], while other approaches using doxorubicin [29,30], vinorelbine [13] or even paclitaxel [16] are emerging.

Finally, electroporation is a flexible tool, however, depending on the cell type or line, the susceptibility of cells to the treatment varies [31], which establishes the greater challenge of the accurate definition of the treatment protocol. For this purpose, the permeabilization curve (permeability increase versus PEF intensity) for the cell type of interest is detected [32]. The detection of cell membrane permeability increase in vitro is usually performed using fluorescent markers [33], however, in vivo the method is hardly applicable. Thus, as an alternative various treatment, prediction models are employed [34] during the treatment planning step [35]. Another option is to use advanced techniques such as magnetic resonance electrical impedance tomography (MREIT) [36]. According to Ampere’s law, the corresponding current distribution map is formed, which is then used to calculate the conductivity maps of the tumor using the MREIT algorithm. Indeed, conductivity changes of the tumor are occurrent during electroporation [37], however, frequently they are hard to distinguish from the conductivity changes associated with the thermal effects [38]. As a result, during the clinical procedures, usually there is no feedback on the efficacy of the treatment and the success rate of the PEF application is unknown. The efficacy of the treatment is analyzed after several days post-treatment, while the evaluation of conductivity changes during the clinical operation might open capabilities to ensure better treatment efficacy and reduce the deviation in terms of successful outcomes. Conductivity change measurement potentially introduces a possibility for real-time feedback in terms of treatment efficiency.

**Figure 1:**
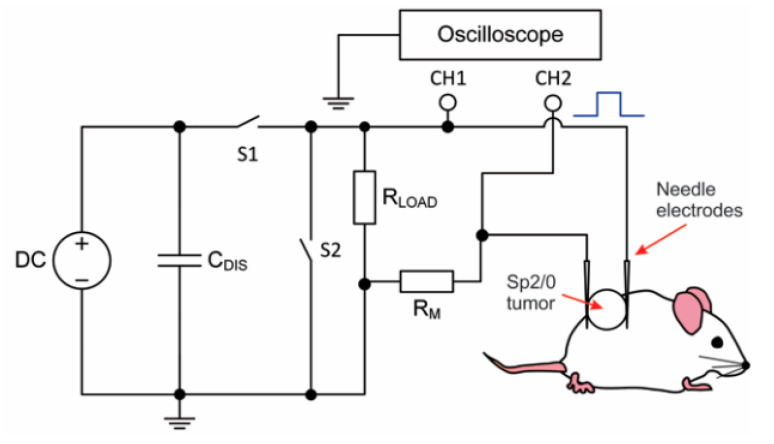
Experimental setup for the electrochemotherapy of tumors, where DC—direct current source, C_DIS_—discharge capacitance, S1/S2 represent the switches, the R_LOAD_ and R_M_ are the load shunting and measurement resistances, respectively; CH1 and CH2—measurement channels of the oscilloscope.

Therefore, in this work, we performed the pilot experiments on doxorubicin-based nano-electrochemotherapy in vivo and compared the results with ESOPE-like treatment. In addition, we performed basic current and voltage measurements to serve as an indicator of successful tumor permeabilization both in the nano and microsecond pulse range. The scheme of the experimental setup is shown in Figure 1.

As a tumor model Sp2/0 cell line was used, while the pulses were delivered via invasive needle electrodes to neglect the influence of skin bioimpedance on the treatment outcome.

## 2. Results

### 2.1. Electroporation of Sp2/0 Cells In Vitro

**Figure 2:**
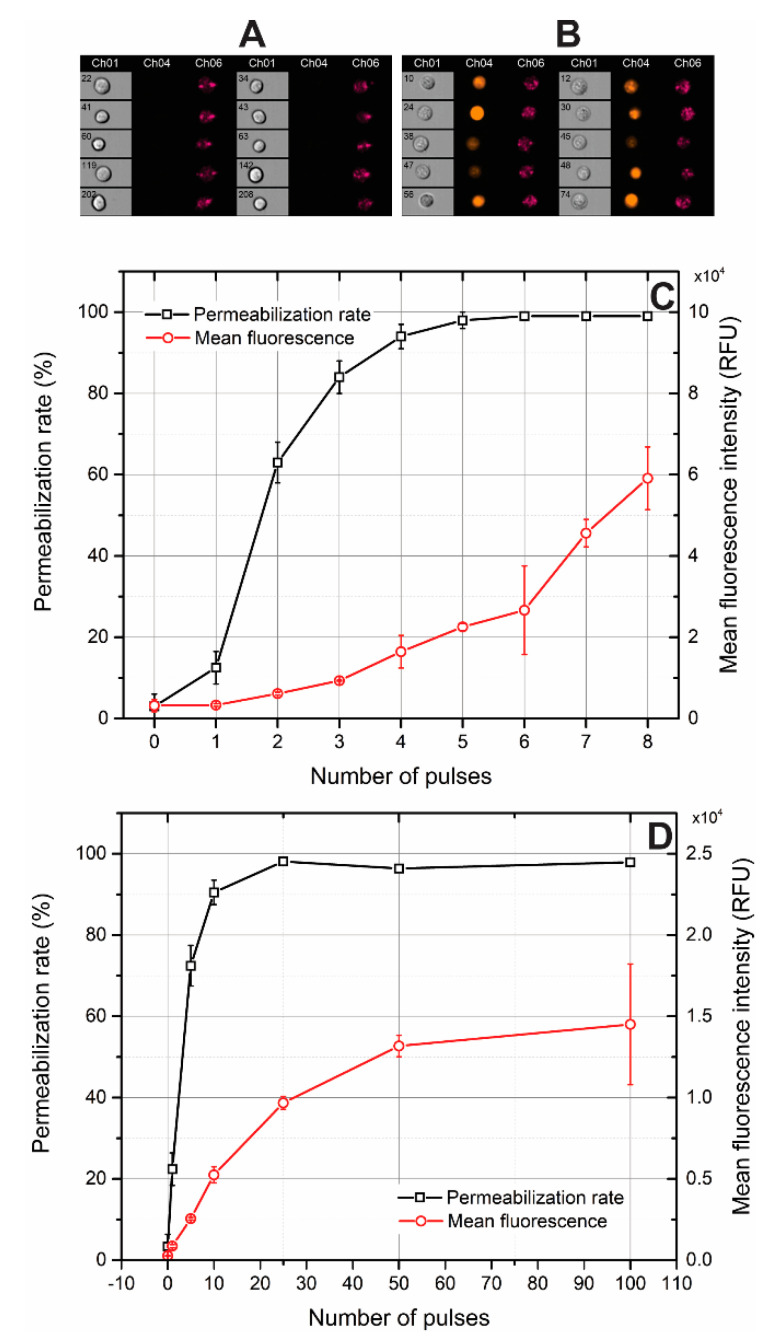
Permeabilization of Sp2/0 cells by pulsed electric fields, where (**A**) photographs of untreated cells; (**B**) photographs of cells treated by 3.5 kV/cm × 800 ns × 100 pulses; (**C**,**D**) permeabilization curves of cells treated by 1.4 kV/cm × 100 µs and 3.5 kV/cm × 800 ns pulsing protocols, respectively. Ch01—brightfield image; Ch04—propidium iodide fluorescence image at wavelength of 488 nm; Ch06—image of forward side scatter.

In order to establish pulsing protocols in terms of the number of pulses and ensure saturated permeabilization, firstly, electroporation was investigated in vitro using propidium iodide (PI) and flow cytometry. The permeabilization curves are presented in Figure 2.

Permeabilized cells featured high fluorescence, while the cells without treatment were impermeable to PI. As it can be in Figure 2C, in the microsecond range, the permeabilization rate of the cells is saturated (>95%) after fourth pulse, however, for nanosecond range pulses (Figure 2D) more than 30 pulses are required. A typical ESOPE protocol involves eight pulses, while for both the nanosecond and microsecond range the electrotransfer efficacy (evaluated as the mean fluorescence intensity, Figure 2C,D) can still be manipulated by the number of pulses. In the case of microsecond pulses, it is 2–3-fold significantly higher both due to the higher energy of the burst and higher electrophoretic component. Therefore, in order to compensate, for the in vivo experiments the number of pulses in the nanosecond protocol was increased to 250 to be comparable to microsecond procedure energy-wise.

### 2.2. Electric Field Distribution and Invasive Electrode Positioning Strategy

**Figure 3:**
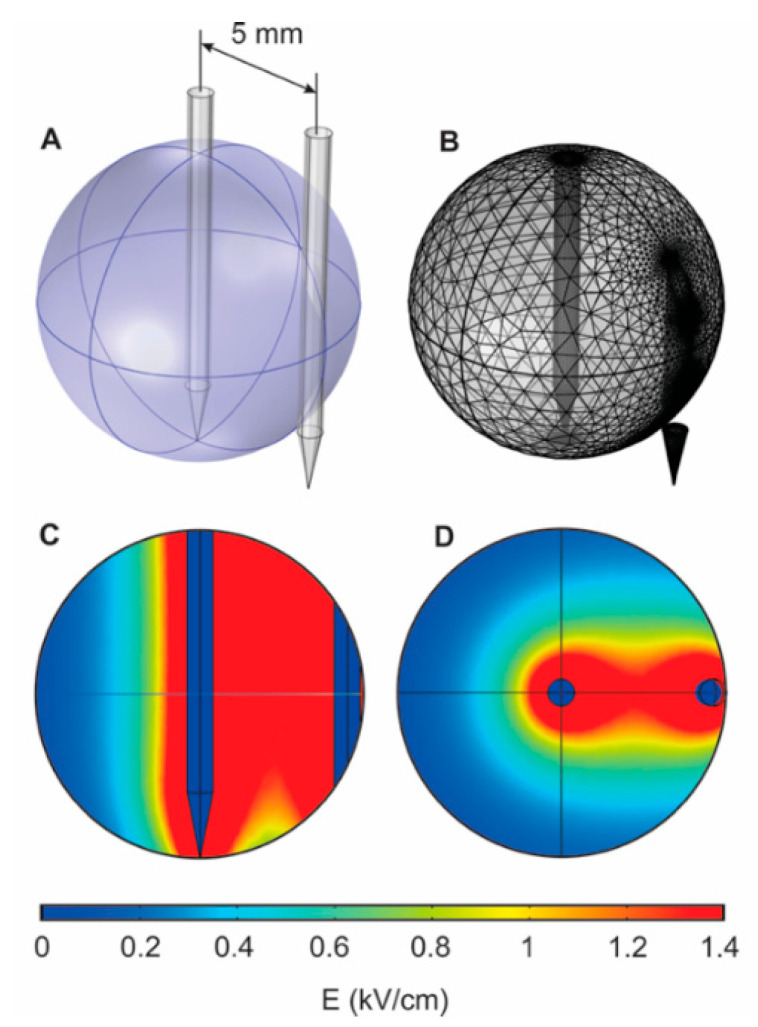
The results of the FEM simulation (1.4 kV/cm pulse), where (**A**) the positioning of the needle electrodes; (**B**) mesh structure; (**C**,**D**) spatial distribution of electric field.

The application of needle electrodes is frequently associated with non-homogeneous electric field distribution, while they are usually applied with bigger tumors compared to parallel plate electrodes. In order to analyze the expected spatial electric field distribution, a primitive finite element method (FEM) model was introduced. The tumor was approximated as a conductive sphere (0.2 S/m) [39] while the needle electrodes pair was injected in the center and on the edge of the tumor, respectively. The results are presented in Figure 3.

It can be seen that the distribution is non-homogeneous, therefore, to compensate and ensure a more homogeneous treatment, the edge electrode was repositioned four times every 90 degrees. As a result of such a strategy, the central part of the tumor receives the higher dose of PEF treatment, which is advantageous. It is important to have less damage of healthy tissue at the tumor interface (edge electrode), while additional necrosis in the central part of the tumor ensures better expected overall treatment outcome and effect localization, which is further confirmed by experiments. The application of the needle electrodes in a conventional way (all electrodes at the edges of the tumor) increases the current demand from the generator, while there are no advantages in terms of field homogeneity or invasiveness of the procedure. It should also be noted that a more complex model should have been introduced if parallel plate electrodes were involved because of the losses of pulse energy in the skin interface. However, it is not the case during invasive procedure and this is a typical approach [40,41], while the main limitation is the approximation of the tumor as a uniform structure, which cannot be controlled experimentally in any way.

**Figure 4:**
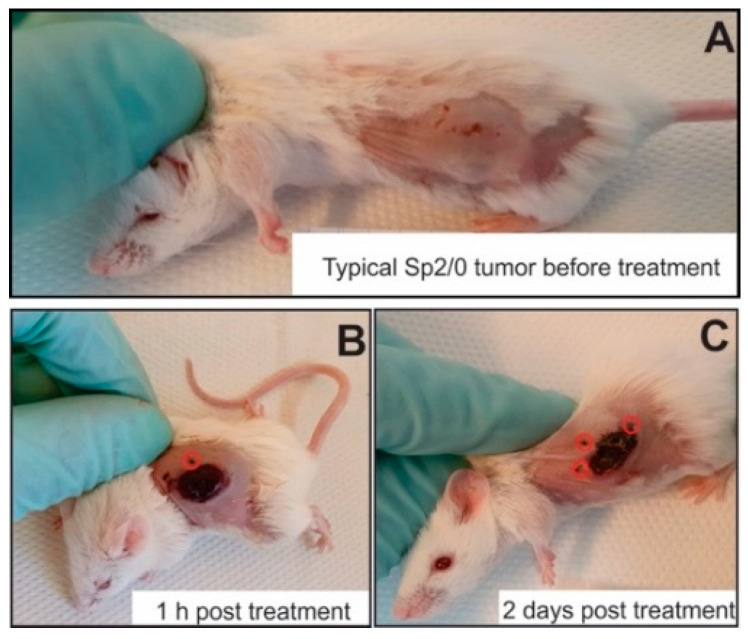
Representative photographs of mice before (**A**) and after 1 h (**B**) and 2 days (**C**) post treatment (3.5 kV/cm × 800 ns × 250 pulses with doxorubicin). The red circles (**B**,**C**) represent visible electrode injection points.

The feasibility of the proposed electrode repositioning strategy was confirmed in vivo. The photographs of treated mice are shown in Figure 4. After pulse application, the change in skin color was detected (Figure 4B) indicating excellent effect localization, while a necrotic tissue was formed 2 days post electrochemotherapy.

It was confirmed that such an electrode repositioning strategy is feasible for electrochemotherapy and ensures a well localized treatment.

### 2.3. Efficacy of Microsecond and Nanosecond Range Electrochemotherapy

**Figure 5:**
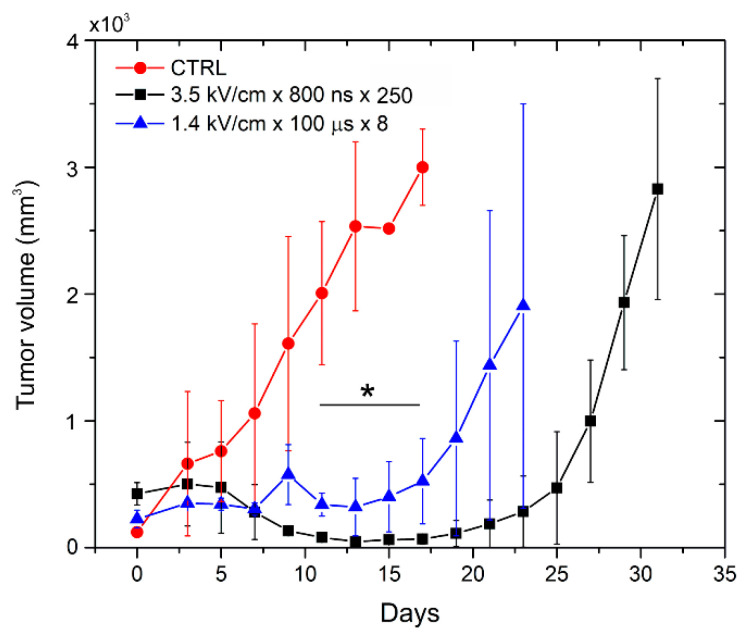
Dynamics of tumor growth after treatment with pulsed electric fields and doxorubicin, where the asterisk corresponds to the statistically significant difference (*p* < 0.05) versus CTRL—untreated control.

During electrochemotherapy, the nanosecond range protocol (3.5 kV/cm × 800 ns × 250) was compared efficacy-wise with a microsecond range procedure (1.4 kV/cm × 100 µs × 8). The growth dynamics of Sp2/0 tumors were evaluated throughout the experiment. The results are summarized in Figure 5.

As can be seen in Figure 5, electrochemotherapy resulted in a significant delay (*p* < 0.05) of tumor growth, while both PEF protocols triggered comparable anticancer efficiency. A tendency of 3.5 kV/cm × 800 ns × 250 pulses being on average more effective than the microsecond range procedure was not statistically significant (*p* > 0.05).

### 2.4. Current and Voltage Waveforms

**Figure 6:**
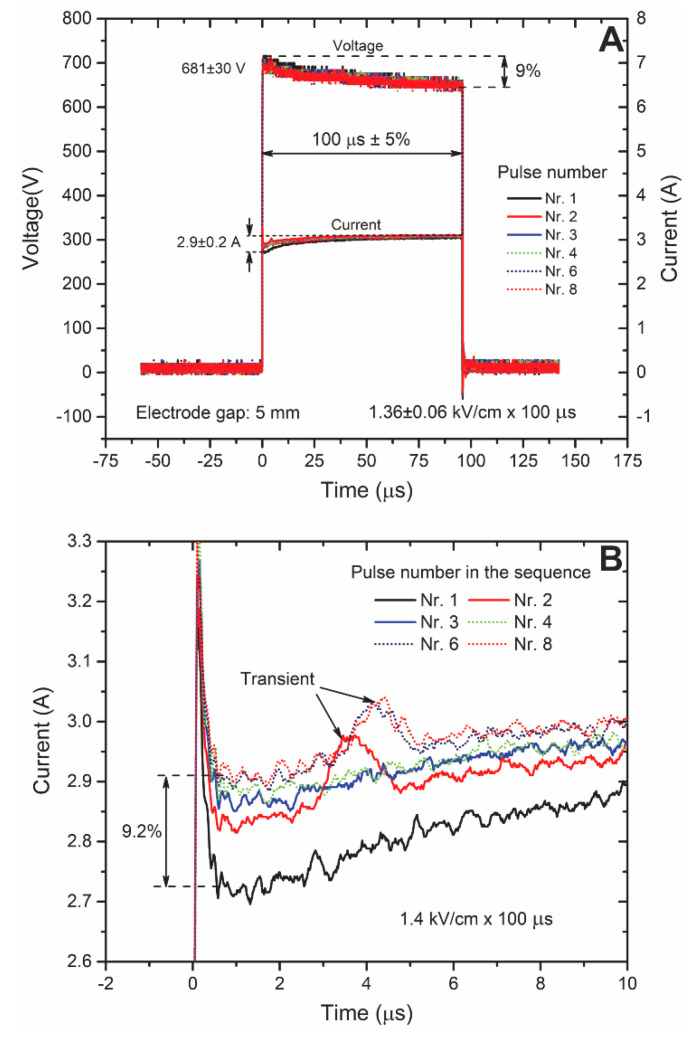
The current and voltage waveforms (**A**) during the microsecond range electroporation and the magnification (**B**) of the current pulses for better informativity.

Current and voltage waveforms were measured during the treatment. The results for microsecond range pulses are shown in Figure 6.

It can be seen (Figure 6A) that a typical voltage droop is occurring, however, it did not exceed 10%, which is within the typical standards for electroporation. As expected, the current was increasing after each pulse (conductivity change), however, the difference also did not exceed 10%, indicating that the thermal influence was negligible. The increase in the current could also be attributed to the increased permeabilization, however, considering the influence of transients on the pulse shape and the small change in the signal (Figure 6B) it was not possible to form conclusions.

**Figure 7:**
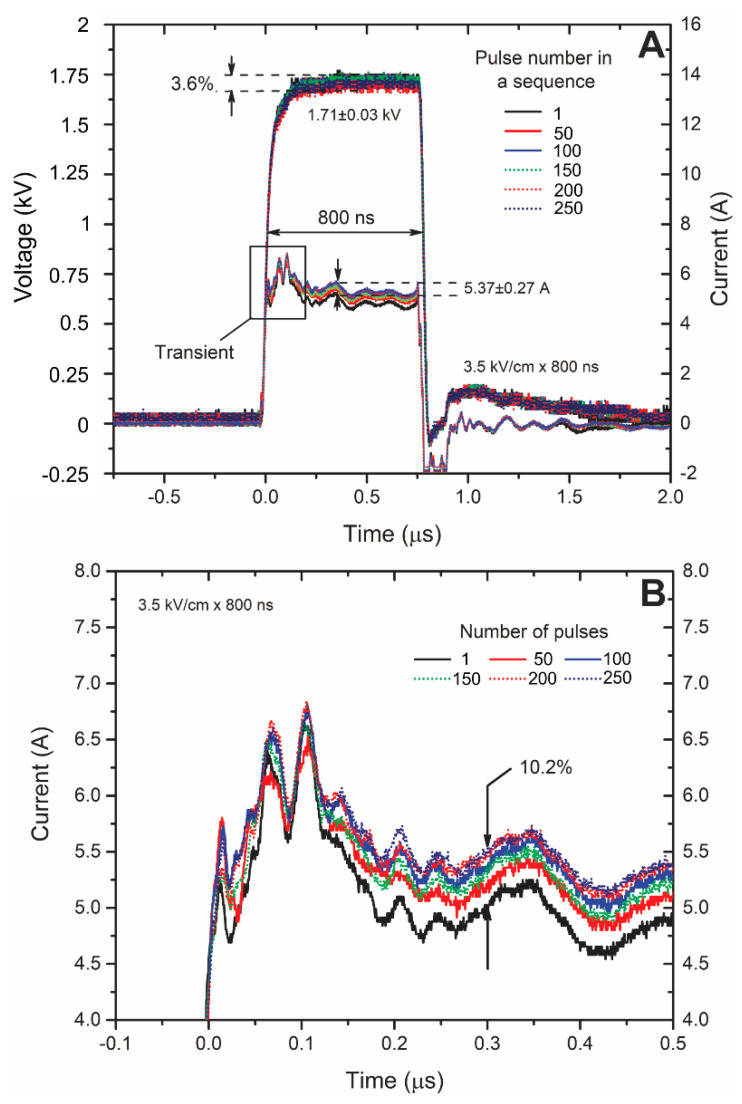
The current and voltage waveforms (**A**) during the nanosecond range electroporation and the magnification (**B**) of the current pulses for better informativity.

A similar tendency was apparent for the nanosecond range pulses (Figure 7). The difference in current amplitude between the first and the last pulse was ~10%. However, the droop of the voltage was smaller due to shorter pulse duration.

The influence of transients was even higher, therefore, in analogy to the microsecond range procedure, it was confirmed that the influence of Joule heating was negligible, while it was not possible to form conclusions regarding the permeabilization state.

Nevertheless, in both cases (microsecond and nanosecond) pulse measurement gives additional information about the treatment and is particularly important if the load impedance is unknown or a high droop of voltage is expected.

Electrical conductivities of biological tissues show frequency-dependent behaviors [42,43,44], therefore, a bioimpedance measurement should be performed using separate RCL measurement devices, while current measurement is useful for the control of delivered energy and gives little to no information about the actual treatment outcome.

## 3. Discussion

Electrochemotherapy is an area, which is currently dominated by microsecond range pulses in combination with bleomycin and cisplatin [10]. However, a nanosecond range electric field can be effectively used to manipulate the permeability of cell membrane and thus improve the drug delivery on the cellular level too.

In this work, we performed a pilot in vivo study to show that doxorubicin can be used in combination with PEF and the nanosecond range pulses can be as effective as the conventional ESOPE protocols. Both protocols, which were used in the study, triggered a statistically significant tumor response, however, parametrically these protocols were hardly comparable. The microsecond range protocol used a 1.4 kV/cm PEF amplitude, which resulted in a ~3 A current in the tumor volume. Also, the duration of pulses exceeded the polarization constant of typical mammalian cells implying that a saturated transmembrane voltage was induced during the pulse. In case of 800 ns pulses, supra-electroporation is triggered, which means that the short duration of the pulse (below polarization constant) requires compensation via the increase in the pulse amplitude [25,45]. Therefore, the 3.5 kV/cm PEF amplitude was selected resulting in a ~5.4 A current in the tumors. Considering that a 250 pulses burst was applied, the total energy of the treatment was ~15% lower compared to the ESOPE protocol.

The current and voltage waveforms were also analyzed throughout the treatment to define the feasibility of pulse measurement for the prediction of the outcome of electrochemotherapy. Indeed, the conductivity-dependent patterns of current increase were apparent with increase in the number of pulses, however, the changes were hardly distinguishable from the characteristic changes in the current due to Joule heating or increased electrolysis [46]. The result is in agreement with established knowledge in the electroporation area [38,47].

Finally, it was confirmed that the proposed invasive electrode positioning strategy is effective for electrochemotherapy and a well controlled localization of the effect can be ensured. Nevertheless, PEF-based techniques can be associated with muscle contractions and pain [48]. However, the application of shorter sub-microsecond range pulses enables better energy control and thus, the better management of side effects. Lastly, shorter pulses are reported to induce a more uniform exposure [49] and could potentially minimize the influence of tissue non-homogeneity [50].

To conclude, nano-electrochemotherapy can be the logical evolution of the currently established ESOPE protocols, offering better energy control and equivalent treatment efficacy. Doxorubicin-based electrochemotherapy, independently on the pulse parameter range, can be an effective alternative to the well-established cisplatin- and bleomycin-based treatments. However, multiparametric analysis is required in the future, including the evaluation of the possible associated side effects [51], which was not covered in this pilot study.

## 4. Materials and Methods

### 4.1. The Pulsed Power Setup

Up to 3 kV, 100 ns—1 ms square wave high voltage and high frequency (up to 1 MHz) pulse generator was used for electroporation [52]. Commercially available electroporation cuvette with 1 mm gap aluminum electrodes (Biorad, Hercules, CA, USA) was used as a load for in vitro experiments. For the in vivo, two stainless steel needle electrodes with a gap of 5 mm were used.

For the current measurement, a shunting resistance of R_M_ = 2.2 Ohm was introduced in series with the electrodes (Figure 1). The voltage drop was measured in parallel with the electrodes and the shunting resistance. Taken that in such a configuration, the voltage will be distributed across R_M_ and the tumor, the exact voltage on the tumor can be recalculated according to the Kirchoff’s law. Measurement directly on the tumor would have required galvanical decoupling of the two signals, which is unnecessary in terms of circuit complexity.

### 4.2. Finite Element Method Simulation

A finite element method (FEM) model of the tumor and needle electrodes was introduced in the study, to assess the spatial distribution of electric field. A 3D triangular mesh model was developed in COMSOL Multiphysics environment (COMSOL, Stockholm, Sweden). The tumor was approximated as a conductive sphere (5 mm radius, 0.2 S/m) and two stainless-steel (0.8 mm diameter, 5 mm gap) needle electrodes were introduced.

### 4.3. Mice and Tumor Induction

BALB/c mice were bred and housed in a mouse facility of State Research Institute Centre for Innovative Medicine, Vilnius, Lithuania. A total of *n* = 12 animals were used. In phosphate-buffered saline (PBS), 1 × 10^6^ of SP2/0 myeloma cells were inoculated under the skin on the back of the 6–8 week old mice. The tumors were allowed to establish and grow until they reached ~150–500 mm^3^ and were ready to treat.

The, 12 mg/kg of doxorubicin (Ebewe pharma, Austria) was injected intraperitoneal 15–20 min prior to the treatment. Two electroporation protocols were employed: (1) 1.4 kV/cm × 100 µs × 8 pulses and (2) 3.5 kV/cm × 800 ns × 250 pulses. After the treatment, the volumes of the tumors were measured by digital caliper every 2–3 days. Tumor volume (mm^3^) was calculated according the formula: V = πlw2/6, where l—length and w—width of the tumor.

All experimental protocols were approved by the Lithuanian State Food and Veterinary Service (approval G2-145) and the study was carried out in strict accordance with the recommendations in the Guide for the Care and Use of Laboratory Animals.

### 4.4. Cell Permeabilization Assay

The permeabilization curve was acquired in vitro. Sp2/0 mouse myeloma cells were cultured in RPMI 1640 medium supplemented with 10 % fetal calf serum, 2 mM glutamine, 100 U/mL penicillin, and 100 μg/mL streptomycin (Gibco, Thermo Fisher Scientific, Waltham, MA, USA) at 37 °C, 5% CO_2_. For the electroporation, the cells were re-suspended at a concentration of 2 × 10^6^ cells/mL in RPMI medium. Seventy microliters (70 μL) of the cell suspension was mixed with propidium iodide (PI, 45 μM) (Sigma-Aldrich, Darmstadt, Germany) fluorescence dye and transferred to the electroporation cuvette. After the pulsing procedure, the cells were stored at room temperature (10 min) for staining. Later, the cells (after pulsing) were transferred to 1.5 mL tubes (Eppendorf, Hamburg, Germany) for analysis by the FlowSight (Amnis, Seattle, WA, USA) flow cytometer. Gate definition and processing were performed in accordance with a previous study [53].

### 4.5. Statistical Analysis

One-way analysis of variance (ANOVA; *p* < 0.05) was used to the compare results. If the ANOVA indicated a statistically significant result (*p* < 0.05), the Tukey HSD multiple comparison test for the evaluation of the difference was used.
